# An ELISA test using a circulating *Mycobacterium bovis* peptide for detecting bovine tuberculosis in dairy cattle

**DOI:** 10.3389/fvets.2024.1384537

**Published:** 2024-05-22

**Authors:** Tawatchai Singhla, Sukolrat Boonyayatra, Nattawooti Sthitmatee, Anucha Sirimalaisuwan, Nitit Maicharoen, Aratchaporn Meemey, Anucha Muenthaisong, Amarin Rittipornlertrak, Srinand Sreevatsan

**Affiliations:** ^1^Faculty of Veterinary Medicine, Chiang Mai University, Chiang Mai, Thailand; ^2^Department of Veterinary Clinical Sciences, College of Veterinary Medicine, Long Island University, Brookville, NY, United States; ^3^Laboratory of Veterinary Vaccine and Biological Products, Faculty of Veterinary Medicine, Chiang Mai University, Chiang Mai, Thailand; ^4^Department of Pathobiology and Diagnostic Investigation, College of Veterinary Medicine, Michigan State University, East Lansing, MI, United States

**Keywords:** bovine tuberculosis, single intradermal tuberculin test, ELISA test, test performance, Bayesian

## Abstract

This study aimed to determine the sensitivity (Se) and specificity (Sp) of a circulating pathogen-specific biomarker (polyketide synthetase 5, Pks5)-based enzyme-linked immunosorbent assay (ELISA) independently or in conjunction with a caudal fold tuberculin (CFT) test for bovine tuberculosis (bTB) screening in dairy cattle. We enrolled 987 dairy cows from 34 herds in Chiang Mai province, Thailand. A conditionally independent Bayesian model with a single population was inferred from the test results. The percentage of positive results for the Pks5-ELISA using 0.4 OD cutoff test and CFT test were 9.0% (89/987) and 10.5% (104/987), respectively. The median of posterior estimates of Se for the Pks5-ELISA test was 90.2% (95% posterior probability interval [PPI] = 76.6–97.4%), while the estimated Sp was slightly higher (median = 92.9, 95% PPI = 91.0–94.5%). The median estimated Se of the CFT test was 85.9% (95% PPI = 72.4–94.6%), while the estimated Sp was higher, with a median of 90.7% (95% PPI = 88.7–92.5%). The posterior estimate for true disease prevalence was 2.4% (95% PPI = 1.2–3.9%). The Pks5-ELISA test yielded characteristics at or above the acceptable standards for bTB detection. Therefore, the pathogen-specific biomarker, Pks5, is a potential detection system for bTB screening and may be applied as an ancillary test together with the currently applied standard method (CFT test) to reinforce the bTB control and eradication programs.

## Introduction

Bovine tuberculosis (bTB), an infectious disease in mammals, is caused by *Mycobacterium bovis* (*M. bovis*), a slow-growing aerobic bacterium that belongs to the *Mycobacterium tuberculosis* complex (1). Cattle are susceptible to the disease; however, other domestic and wild animals, including humans, can also be infected with the pathogen. Over 50 million cattle are purportedly infected with *M. bovis* globally, resulting in annual economic losses of approximately 3 billion USD (2). Moreover, bTB can spread to humans as a neglected zoonotic disease and kill people every year (1). In 2017, the number of new cases of *M. bovis* infection in humans reported by the World Health Organization was 142,000, with over 12,500 deaths (3).

bTB is characterized by a chronic and slowly progressive disease with nonspecific clinical signs. In early stage of infection, most infected animals do not exhibit any clinical signs of the disease. However, the infected animals can shed the pathogen to healthy animals during this period (1). Therefore, early detection by (or a combination of) high-accuracy diagnostic methods and elimination of reactors play an important role in the success of bTB control and eradication programs. Normally, bTB is diagnosed by indirect methods that interrogate host immune responses (cell mediated immune response), such as a caudal fold tuberculin (CFT) test and gamma interferon release assay (4, 5). In cattle, the CFT test is usually performed at the caudal fold of the tail by intradermally injecting bovine purified protein derivative (PPD) (5). While CFT is easy to perform in the field, it is labor-intensive and inaccurate (6). Cross-reactions with other environmental or saprophytic Mycobacteria have been reported, leading to false-positive results (5). Moreover, CFT purportedly also has a poor ability to detect infected animals (7) because several studies have reported varying levels of sensitivity (Se) and specificity (Sp) for CFT (6). For instance, a study in Thailand reported the Se and Sp of the CFT test, ranging from 43.2 to 80.2% and from 82.9 to 97.6%, respectively, while a study in the Uruguay reported that the Se and Sp of the test ranged from 56.8 to 98.9% and from 34.2 to 95.5%, respectively (6, 8).

Several researchers have tried to develop diagnostic techniques for bTB detection to limitations of a current available diagnostic test. A US study reported that pathogen-specific peptides released into the blood circulation of cattle during *M. bovis* infection, including polyketide synthetase 5 (Pks5) were amenable to testing subclinical animals (7). An enzyme-linked immunosorbent assay (ELISA) has been developed for Pks5 as a biomarker for bTB diagnosis and has exhibited high accuracy, with Se and Sp ranged from 79.0 to 100.0% and from 75.0 to 95.0%, respectively. Furthermore, this peptide can minimize the cross-reactivity between *M. bovis* and other mycobacterial infections and distinguish between *M. bovis*-infected and *M. bovis*-exposed animals (7). Additionally, the advantages of the Pks5 detection test are that it is inexpensive, available in large samples, and not time consuming. However, this technique has not been performed under field conditions on a large population or in different geographical regions with variable disease situations and management practices.

In general, Se and Sp of a diagnostic test are estimated by comparison to a gold standard or a reference test. However, in some situations, the gold standards for testing of many diseases are scarce. To solve this problem, a latent class analysis has been increasingly used to determine an accuracy of a screening test when a gold standard is lacking or a reference test is imperfect (9, 10). Recently, a Bayesian latent class analysis has been used to estimated characteristics of various bTB diagnostic tests in cattle without the comparison of a gold standard and the true disease prevalence is unknown (6, 10).

This study aimed to evaluate the sensitivity and specificity of a Pks5-ELISA test in comparison to the caudal fold tuberculin test through the Bayesian latent class model, in diagnosing bTB in dairy herds.

## Materials and methods

### Study area

This study included 34 dairy farms located in three districts of Chiang Mai province, Thailand, including Mae Wang, Doi Lo, and San Pa Tong Districts. All herds were smallholder dairy farms with an average herd size of 63 (min-max = 12–126) heads. According to the data obtained from the Chiang Mai provincial livestock office, 12 of these 34 herds were identified as CFT-positive herds based on presenting of at least one reactor in the herd during the period 2018 to 2020. The 22 remaining herds were from farms that had no CFT-positive animals during the same period.

### Caudal fold tuberculin test

In total, 987 dairy cows (age ≥ 1 year) from the 34 herds were tested for bTB using the CFT test by an author or staff of the Chiang Mai provincial livestock office between March and May 2020 as a part of the annual bTB screening program provided by the Chiang Mai provincial livestock office. For CFT testing, the skin thickness of the caudal folds at the injection site was measured using calipers before inoculation. After that, Bovine PPD of 0.1 mL or 2,000 IU (Bovituber® PPD, Synbiotics, Lyon, France) was injected into the caudal fold of the tail. At 72 h after inoculation, the test results were determined using calipers to measure an increase in skinfold thickness and palpation by the same researcher and interpreted following the World Organization for Animal Health (WOAH) Terrestrial Manual for Mammalian tuberculosis (11). The outcomes were considered reactor (positive) when any swelling, sensitivity, or increase in skin thickness was detected at the inoculation site.

### Indirect ELISA for Pks5 detection

A total of 987 serum samples were collected from 34 dairy herds for the Pks5-ELISA test following a previously described protocol (7). Positive control sera were obtained from CFT test- and gamma interferon releasing assay-positive dairy cattle and confirmed by macroscopic lesions, histological lesions (with acid-fast staining), and PCR. In addition, laboratory-grade fetal bovine serum samples were used as negative controls. Briefly, 0.05 M carbonate–bicarbonate buffer (coating buffer, pH 9.6) was used for individual dilution of dairy cow sera and positive- and negative-control sera at a 1:200 dilution. Each well of SPL Maxibinding polystyrene flat-bottom immunoplate was coated with 100 μL of the diluted samples. The samples were plated in triplicate and incubated overnight at 4°C. After three washes with 200 μL/well of PBS containing 0.05% TW20 (PBS-T), the plates were blocked with 150 μL/well of 1% skim milk PBS-T buffer at RT for 1 h. Then, the plates were washed thrice with 200 μL/well of PBS-T and incubated with 100 μL/well of a 1:10,000 dilution of Pks5 resuspended (monoclonal antibodies against Pks5) in a 1% solution of skim milk PBS-T at RT for 1 h. Following three washes, the plates were incubated with 100 μL/well of affinity purified antibody peroxidase-labeled goat anti-mouse IgG (H + L) [Kirkegaard-Perry Laboratories (KPL), Marylan, US] diluted 1:1,000 in 1% solution of skim milk for 1 h at RT, followed by another wash. ELISA plates were performed with 100 μL/well of 3,3′,5,5′-tetramethylbenzidine (TMB) (KPL TMB Microwell peroxidase substrate system) and incubated at RT for 15 min in the dark. Finally, the color reaction was stopped by adding 50 μL/well of 2 M sulfuric acid, and the optical density (OD) at 450 nm was recorded using a M965+ microplate reader and software (Metertech Inc., Taipei, Taiwan). To interpret the test results, four different cutoff values (≥0.2, ≥0.3, ≥0.4, and ≥ 0.5 OD) were used to distinguish positive from negative samples (7).

### Sensitivity and specificity estimation

Cohen’s kappa analysis was performed to assess the agreement between the test outcomes of the CFT and ELISA tests. The agreement was classified into six categories based on kappa values ranging from 0 to 1 including slight (0–0.20), fair (0.21–0.40), moderate (0.41–0.60), substantial (0.61–0.80), and almost perfect (0.80–1.0) agreement (12).

Bayesian latent class analysis was performed to estimate the Se and Sp of the CFT test and Pks5-ELISA test (separately at each cutoff level). The CFT for bTB diagnosis is based on the principle of detecting a CMI response, while the Pks5-ELISA test is based on the pathogen-specific peptide (Pks5) detection. Thus, conditional independence was assumed for the test results. The two tests were applied to dairy herds raised in the same region, where the feed resources and management practices were similar. Therefore, the tests can be assumed to have been implemented in a single population, as proposed in previous studies (6, 13). Thus, a Bayesian modeling of two conditionally independent with one population was modeled to estimate the Se and Sp of both tests as well as the true disease prevalence. The Bayesian latent class analysis was performed under the *k* populations, and the counts (*Y_k_*) of the different combinations of test results, including +/+, +/−, −/+, and −/−, followed a multinomial distribution:


$$Y_{k}\;|\;P_{qrk}∼\mathrm{multinomial}\;\left(n_{k},\left\{P_{qrk}\right\}\right)$$

where *qr*. is the multinomial cell probability for the two-test outcome combination, and *P_qrk_* is a vector of probabilities of observing the individual combinations of the test results.

The prior distributions of Se and Sp for the CFT and ELISA tests and the disease prevalence were created from previous reports and modeled as beta distributions, as shown in Table 1 (6–8, 14). The published study means of the central values were used for the most likely value selection, whereas the lowest modal value was used as a 95% lower limit of the prior distributions. All analyses were implemented in JAGS 4.3.0, using the rjags and R2jags packages in R version 4.1.2 (15–17). The first 10,000 iterations were omitted as the burn-in phase, and the posterior distributions were computed after 100,000 iterations of the models.

**Table 1:** Priors estimated for sensitivity and specificity of the ELISA test, the CFT test, and the disease prevalence.

Diagnostic tests	Parameters	Mean	95% CI^a^	References
Pks5-ELISA^b^	Sensitivity	92.0	> 79.0	Lamont et al (7)
	Specificity	93.0	> 75.0	Lamont et al (7)
CFT^c^	Sensitivity	87.6	> 75.3	Singhla et al. (6), Picasso-Risso et al. (8)
	Specificity	83.6	> 74.2	Singhla et al. (6), Picasso-Risso et al. (8)
	Prevalence	2.0	< 8.0	Picasso-Risso et al. (8), Nuamjit and Rodtian (14)

a 95% confidence interval.

b Pks5-based enzyme-linked immunosorbent assay.

c caudal fold tuberculin test.

For model convergence testing, the Gelman-Rubin diagnostic plots were visually inspected by running multiple chains from different starting values (18). The influence of prior distributions on posterior estimates of all model parameters was assessed using sensitivity analysis. Each prior value was replaced by uniform prior on the range from 0 to 1 (9, 19). In addition, the assumption of conditional independence between the two tests was validated by comparing the DICs between the models with and without the covariance term (9, 19).

## Results

### Results from the diagnostic tests

Of the 987 dairy cows, 104 and 883 were identified as positive and negative by the CFT test. For the Pks5-ELISA test, 477, 200, 89, and 46 dairy cows were identified as positive by the OD cutoff values of ≥0.2, ≥0.3, ≥0.4, and ≥ 0.5, respectively; whereas, 510, 787, 898, and 941 dairy cows were identified as negative by the OD cutoff values of ≥0.2, ≥0.3, ≥0.4, and ≥ 0.5, respectively. All the outcomes of both tests are shown in Table 2. For the agreement analysis, the CFT test and all OD cutoff ELISA test results exhibited slight agreements with the kappa values ranged from 0.08 to 0.17.

**Table 2:** Cross-classified results from the CFT^a^ test and the Pks5-ELISA^b^ test performing in 987 dairy cattle for bovine tuberculosis diagnosis.

Diagnostic test results		CFT^a^		Total
		Positive	Negative	
Pks5-ELISA^b^ OD ≥0.2	Positive	68	409	477
	Negative	36	474	510
	Total	104	883	987
Pks5-ELISA^b^ OD ≥0.3	Positive	42	158	200
	Negative	62	725	787
	Total	104	883	987
Pks5-ELISA^b^ OD ≥0.4	Positive	24	65	89
	Negative	80	818	898
	Total	104	883	987
Pks5-ELISA^b^ OD ≥0.5	Positive	13	33	46
	Negative	91	850	941
	Total	104	883	987

a caudal fold tuberculin test.

b Pks5-enzyme-linked immunosorbent assay.

### Bayesian model

The Se estimates for the Pks5-ELISA test were highest when the ≥0.2 OD cutoff was used in CFT test model, with medians of 91.7% (95% PPI = 80.4–97.8). However, the Se of this test was consecutively reduced by increasing of the OD cutoff values, with the median values ranged from 89.8 to 91.7%. On the other hand, the estimated Sp for the Pks5-ELISA was lowest when the ≥0.2 OD cutoff was used in CFT test model with the median of 54.2% (95% PPI = 50.9–57.5%). The Sp estimates for the test had a direct relationship with the cutoff values, with the median values ranged from 54.2 to 96.8%.

The medians of posterior estimates for Se of the CFT test ranged between 85.7 and 86.2%. On the other hand, the medians of posterior estimates for Sp of the CFT test ranged between 90.0 and 92.0%. However, estimated true disease prevalence was highest when the Pks5-ELISA using ≥0.2 OD cutoff value was performed, with the median of 3.9% (95% PPI = 1.6–6.7%) in comparison with the CFT test. The posterior estimates of both diagnostic tests and the true disease prevalence are shown in Table 3.

**Table 3:** Posterior estimates for sensitivity and specificity of the ELISA^a^ test using four different cutoffs, the CFT^b^ test, and the disease prevalence.

Diagnostic tests	Parameters	Median (%)	95% PPI^c^ (%)
Pks5-ELISA^a^ OD ≥0.2	Sensitivity	91.7	80.4–97.8
	Specificity	54.2	50.9–57.5
CFT^b^	Sensitivity	86.2	72.7–94.7
	Specificity	92.0	89.5–94.3
	Prevalence	3.9	1.6–6.7
Pks5-ELISA^a^ OD ≥0.3	Sensitivity	90.9	78.5–97.6
	Specificity	82.5	79.8–85.0
CFT^b^	Sensitivity	86.1	72.7–94.7
	Specificity	91.7	89.6–93.5
	Prevalence	3.5	2.0–5.6
Pks5-ELISA^a^ OD ≥0.4	Sensitivity	90.2	76.6–97.4
	Specificity	92.9	91.0–94.5
CFT^b^	Sensitivity	85.9	72.4–94.6
	Specificity	90.7	88.7–92.5
	Prevalence	2.4	1.2–3.9
Pks5-ELISA^a^ OD ≥0.5	Sensitivity	89.8	75.7–97.3
	Specificity	96.3	94.9–97.5
CFT^b^	Sensitivity	85.7	72.0–74.5
	Specificity	90.0	88.0–91.7
	Prevalence	1.4	0.6–2.6

a Pks5-enzyme-linked immunosorbent assay.

b caudal fold tuberculin test.

c 95% PPI = 95% posterior probability interval.

The final model was verified to exhibit proper convergence after visually inspecting a Gelman-Rubin diagnostic plots, and autocorrelation was eliminated after burning-phase by omitting the first 10,000 iterations. For the sensitivity analyses of the final model, there was no appreciable effect (the median value change >25%) in the posterior estimates for the Se and Sp of the Pks5-ELISA using ≥0.2, ≥0.3, and ≥ 0.4 OD cutoffs, and for the Se and Sp of the CFT test after the priors of any parameter were replaced by non-informative distributions. For instance, estimated Sp of Pks5-ELISA using ≥0.4 OD cutoff changed only 0.1% (from 92.9 to 92.8%) after non-informative distribution was applied. This indicates the robustness of the model. However, a larger change in posterior estimates for the Pks5-ELISA Se, CFT Se, and the true disease prevalence was observed in the models of Pks5-ELISA using ≥0.5 OD cutoffs in comparison with the CFT (from 89.8 to 60.0%, from 85.7 to 58.6%, and from 1.4 to 2.0%, respectively). This finding suggests that prior information of these parameters has a strong effect in the model.

According to the assumption of the model, the conditionally independent model, without a covariance term between the CFT and ELISA (all OD cutoff values), mostly showed slightly lower the DIC values than the conditionally dependent model. However, both tests follow different principles in diagnosing bTB, as previously described. Therefore, a conditional independent model was preferred as the final model.

## Discussion

The present study determined the performance of the Pks5-ELISA using the circulating pathogen peptide (Pks5) as a biomarker for bTB diagnosis, and CFT test which is routinely used for diagnostic tests in bTB control and eradication programs under Thai conditions using a Bayesian latent class analysis. For Bayesian modeling, the analysis was performed using a one-population model because the two tests were implemented in dairy herds located in the same area and followed similar animal breed, feed resources and management practices. Thus, all animals were considered a single population, as assumed in previous studies (6, 10).

For the test results, the slight agreement between both tests was consistent with the low conditional dependence between the ELISA and CFT tests in both infected and non-infected animals when the conditionally dependent model was analyzed for model assumption testing. These findings together with the scientific principles of these two diagnostic tests, ELISA and CFT are two independent tests. Application of using ELISA and CFT together as multiple tests for the diagnosis of bTB can be beneficial, especially when they are applied as parallel tests, which can increase the sensitivity of the bTB diagnosis in current bTB control and eradication programs (6, 20).

Regarding the characteristics of the ELISA test in comparison with CFT tests, its posterior estimates for Se in all OD cutoff values were higher than those of the CFT test, whereas its Sp were lower for the cutoff value of ≥0.2 and ≥ 0.3 and higher for the cutoff value of ≥0.4 and ≥ 0.5. Thus, lowering the OD cutoffs for the ELISA test could increase the Se of the test, whereas false positive results could be increasingly occurred leading to the lower Sp of the ELLSA test using ≥0.2 and ≥ 0.3 OD cutoffs. However, the characteristics of the ELISA test using ≥0.2 and ≥ 0.3 OD cutoffs were lower than those reported in a previous study in the US that reported Se and Sp values ranged from 99 to 100% and from 75 to 90.0%, respectively (7). These OD cutoff values have been suggested for a high disease prevalence setting, whereas the serum samples of this study were from the low disease prevalence areas. This may affect Bayesian inference of the characteristic estimation for the ELISA test. On the other hand, posterior estimates for the ELISA test using ≥0.4 and ≥ 0.5 OD cutoffs are close to the previous report which suggested that these cutoff values were suitable for low-bTB-prevalence regions by minimizing the cross-reactivity with other mycobacterial infections and exhibiting high Sp (7). Estimates for the true disease prevalence were negatively associated with the OD cutoffs. This consistent with the Se of the Pks5-ELISA test, as described previously. Furthermore, the true disease prevalences were close to those reported by a 2022 study in Thailand ranged from 1.4 to 7.8% (10). Conversely, a study in northern Thailand using a Bayesian approach reported a higher prevalence, ranging from 14 to 22% (6); however, it only assessed bTB-infected herds, while the samples of the present study were obtained from both infected and uninfected herds. This may explain the differences in the disease prevalence estimates.

For the CFT, the estimated Se was close to those reported in USA in 2004 (84.9–93.02%) (21) and Thailand in 2019 (87.6%) (6). The posterior estimates for CFT-test Sp in the present study were similar to those reported by a study in Thailand in 2019 (6) and a meta-analysis in the UK and Ireland in 2018 (22). However, the CFT-test Sp was slightly higher than that in previous studies in low-prevalence areas, which reported Sp values ranging from 83.6 to 90.6% (23, 24). Several reports have suggested that the size of both the CFT-positive results at animal skins and bTB gross lesions is positively related to the stage of infection (4, 25). In Thailand, the test and slaughter policy for the bTB eradication program has been applied to dairy cattle since 2012. Therefore, CFT-reactors are continually removed from infected herds every year, and cattle with advanced stage of bTB are uncommon. This could affect the Se and Sp values determined by the CFT test in the Bayesian model. However, CFT-positive animals are rarely tested for confirmation or surveillance at slaughterhouses in Thailand.

Several countries have implemented bTB eradication programs using a test and slaughter policy. Nevertheless, bTB is still reported in several countries, although the disease prevalence is quite low in some countries including Thailand (10, 26–29). This may be due to the imperfect performance of bTB screening tests such as the CFT test and the slow progression (and long subclinical phase) of the disease (4, 29). The current study reports the high sensitivity and specificity of the ELISA test using Pks5 as a biomarker for bTB diagnostic testing. The advantage of Pks5-ELISA test is not only that it is a high-throughput laboratory test, but also the fact that different OD cutoffs can be selected for appropriate interpretations in regions with different prevalence (7). In contrast, the CFT test is a labor intensive techniques that requires at least two farm visits and animal restraints for performing the test and observing the test reaction. It also requires experienced experts to perform and interpret results. The staffs should be trained before performing in a field with a precise measuring instrument such as calipers as same as the current study. Moreover, ELISA, which directly detects pathogen peptides, can be repeated multiple times without the limitation of testing frequency. However, the CFT test, which detects CMI, cannot be repeatedly performed in animals within a period shorter than 60 days according to the occurrence of a false negative result from desensitization (30–32). Thus, the limitations of the CFT test may interfere with or prolong bTB eradication programs. Therefore, the Pks5-ELISA test can serve as a useful ancillary test together with the currently available official bTB screening test (the caudal fold tuberculin test) in Thailand to reinforce the disease control and eradication programs in the country.

## Conclusion

We have discussed the characteristics of the CFT test as the current standard test for bTB diagnosis in Thailand and an ELISA test using the circulating pathogen peptide (Pks5) as a biomarker for bTB detection in dairy cows under field conditions using a Bayesian latent class analysis. The sensitivity and specificity of the ELISA are suitable for bTB detection. Therefore, our results highlight an option for the Pks5 ELISA to be used as an ancillary test with the standard test for bTB screening to improve the efficiency of bTB control and eradication program. However, the ELISA was implemented only in dairy cows raised in low-bTB-prevalence areas. Thus, future studies must be conducted in other populations with different disease prevalence levels or compared with more specificity tests such as an intradermal comparative cervical tuberculin skin test to achieve more information.

## Data availability statement

The raw data supporting the conclusions of this article will be made available by the authors, without undue reservation.

## Ethics statement

The study was approved by the Institutional Review Board (or Ethics Committee) of the Faculty of Veterinary Medicine Chiang Mai University, the Animal Care and Use Committee (protocol code S26/2564). The studies were conducted in accordance with the local legislation and institutional requirements. Written informed consent was obtained from the owners for the participation of their animals in this study.

## Author contributions

TS: Formal analysis, Investigation, Methodology, Writing – original draft, Writing – review & editing. SB: Conceptualization, Supervision, Writing – review & editing. NS: Resources, Writing – original draft. AS: Conceptualization, Writing – original draft. NM: Writing – original draft. AMe: Investigation, Writing – original draft. AMu: Investigation, Writing – original draft. AR: Investigation, Writing – original draft. SS: Conceptualization, Supervision, Writing – original draft.
